# Drug behaviors, sexually transmitted infection prevention, and sexual consent during chemsex: insights generated in the Budd app after each chemsex session

**DOI:** 10.3389/fpubh.2023.1160087

**Published:** 2023-05-18

**Authors:** Tom Platteau, Corinne Herrijgers, Eric Florence, Karolien Poels, Peter Verboon, Ludwig Apers, Heidi Vandebosch

**Affiliations:** ^1^Department of Clinical Sciences, Institute of Tropical Medicine, Antwerp, Belgium; ^2^Department of General Internal Medicine, Infectious Diseases and Tropical Medicine, Antwerp University Hospital, Edegem, Belgium; ^3^Department of Communication Studies, University of Antwerp, Antwerp, Belgium; ^4^Faculty of Psychology, Open University, Heerlen, Netherlands

**Keywords:** chemsex, drug use, sexual health, STI prevention, sexual consent

## Abstract

Chemsex refers to the intentional use of drugs before or during sex in a specific context, typically involving prolonged sex sessions with multiple partners. Engaging in chemsex is associated with a wide range of health risks and related risk behaviors. We developed a mobile phone application (‘Budd-app’) to support and inform chemsex participants, reduce potential negative impacts associated with chemsex (e.g., physical, psychological and social health harms), and encourage more reasoned participation. During Budd’s development process, 11 participants completed a survey after each chemsex session they attended. This data collection approach provided precise experiences on drug related behavior, prevention measures for sexually transmitted infection and sexual consent on 63 chemsex sessions. The mean duration of chemsex sessions was 17.5 h. Polydrug use was reported during 95% of chemsex sessions with an average of 3.5 agents per session. Unsafe dosing occurred at 49% of chemsex sessions, and 9/11 participants dosed unsafely at least once. Seven participants did not consistently take measures to prevent STI transmission. Nine had experienced peer pressure, both regarding drug use and sexual health. The same number reported sex without consent, not respecting others’ boundaries as well as their own boundaries not being respected. Many participants experienced negative impact of their chemsex behavior during (7/9) and after (8/9) chemsex. Through participants’ behavior assessment during multiple chemsex sessions, ‘within-person’ variability can be clarified. This clarification provides valuable insights in personal, emotional and contextual vulnerabilities. These insights can direct an individualized care and support trajectory aimed at addressing those vulnerabilities.

## 1. Introduction

The term chemsex refers to the use of psychoactive substances—such as methamphetamine, γ-hydroxybutyrate (GHB), γ-butyrolactone (GBL), or mephedrone—in sexualized settings (1). Although the practice of sexualized drug use has been observed across a diversity of people (2), chemsex is primarily observed among gay, bisexual, and other men who have sex with men (GBMSM) (1). Chemsex is characterized by its specific context, with the use of digital technologies to meet for sessions that may last for several days. Moreover, GBMSM who engage in chemsex experience adverse impact from their chemsex use (3). Throughout this paper we will use the term ‘chemsex participants’ to refer to GBMSM who engage in chemsex in an effort to increase readability.

In scientific literature, there is no consensus definition of the term ‘chemsex’, in terms of substances used. Substances can be categorized by toxicology, or their effect during sex. As not all drug use during sex is considered chemsex, Strong et al. categorized drugs used in a sexual context into three distinct categories (4). The first category consists of ‘universally’ considered chemsex drugs, such as metamphetamine, GHB/GBL and mephedrone (“reported in many studies … triggering a particular intensity of sexual interaction that is qualitatively different from other drugs included in the table”; p. e717). The second category contains chemsex drugs in some regions but not in others, such as ketamine, cocaine and MDMA (“drugs that are considered part of the chemsex scene in some countries,” p. e717). The third category consists of drugs that are consumed by users during chemsex events, but that are not considered chemsex drugs, such as poppers, marijuana, and erectile dysfunction drugs (“substances commonly used alongside, but not typically constituting, chemsex drugs themselves”; p. e717).

The 2017 European Internet Survey has shown that 11 % of Belgian male respondents who reported to have sex with men (MSM), have used stimulant drugs in the past four weeks to make sex more intense or last longer (5). Beside, over 40 % (40.63%) of 1,549 chemsex participants in four European countries reported ‘unwanted side effects’ of their chemsex use (6). Lastly, substantial increases in chemsex related deaths are observed in France and the UK (7, 8).

In July 2022, the World Health Organization (WHO) highlighted the need for novel and tailored approaches to the growing phenomenon of chemsex (9). In answer to that, we developed a mobile phone application (‘app’) with the aim to improve care and support for chemsex participants by facilitating harm reduction strategies. The app, called ‘Budd’, was developed using the Intervention Mapping Protocol (IMP), in co-design with stakeholders and chemsex participants and was launched in April 2022. A dedicated article describing the three-year process of development was published elsewhere (10).

During Budd’s development process, chemsex participants were consulted three times. During a first consultation round, twenty chemsex participants provided insight in their needs and adhered risk reduction practices (before, during and after a chemsex session) via in-depth interviews (11). The identified needs could be translated into content and features in the app to optimize its relevance. Adhered risk reduction practices were integrated as app components in order to improve the use and acceptability of the app. A second consultation round was organized among eight chemsex participants to pilot-test the proof of concept version of the app on usability and acceptability (10). In a third consultation, prior to launching the app, 11 chemsex users tested the final version of the app on its effectiveness. The results of this effectiveness study will be submitted for publication later.

During their eight-week participation in this effectiveness study, participants filled out a survey on their chemsex related behavior after each chemsex session they attended. The analysis of these data is presented in the current manuscript, implying that this is a sub-analysis using data collected during the effectiveness study. The data provide very concrete information per session and per participant. This may complement the perspective of interviews or cross-sectional survey studies, where people reflect and describe their behavior over a period of time, following a chemsex event. Results stemming from studies using these qualitative (interviews) and cross-sectional study designs are potentially very interesting yet limited in their validity by recall bias that may occur. The goal of this paper is to describe very precisely chemsex participants’ experiences with drug use, STI prevention and sexual consent when participating in chemsex sessions.

## 2. Methods

### 2.1. Study design

The data presented below describe participants’ behavior during chemsex sessions before and during their access to the Budd-app, regardless their use of the app. Therefore, for the data-analysis presented here, case study or descriptive analysis may fit the design. The overarching effectiveness study however had a single case design with the introduction of an intervention (access to the Budd-app) during the study period. Using a single-case design is nowadays becoming more common in psychology, to study behavior and behavior change (12). It is characterized by the systematic and repeated measurement of a dependent variable in a small group of participants. It is especially useful in small or heterogeneous populations (where a randomized controlled trial is not feasible), for behavior that does not occur very often and/or for intervention studies with a limited study period, as it provides insights in the personal use and effect per participant in a relatively small sample size. Therefore, a single case design seemed the most appropriate way to describe drug behaviors, STI prevention and sexual consent during chemsex sessions.

### 2.2. Study setting

The study was conducted at the Institute of Tropical Medicine (ITM) in Antwerp, Belgium. The outpatient clinic at ITM consists of an HIV treatment center, PrEP consultation and low-threshold HIV/STI testing center. Participants were recruited via these channels. Although participants needed to visit the clinic for enrolment, the data we present here were collected during the study period, and were self-collected in the app. Therefore, a specific visit to the clinic was not required to fill in the information.

### 2.3. Study participants

GBMSM who reported to engage in chemsex were eligible for participation. Additional inclusion criteria were age above 18 years, identification as member of the LGBTQ+ community, intentional combination sex and drugs in the previous two months, understanding English or Dutch well, and owning a smartphone.

A convenience sample of patients attending ITM’s HIV/STI clinic were approached to participate in the study. Participants were selected by health care providers who consult in the clinic. Additionally, a leaflet was sent out via social media (by Sensoa, the Flemish center of expertise on sexual health); no participants were recruited via the latter approach. In order to generate solid results from our SCD, we aimed to include 10 participants for the full study period. Twelve participants had been enrolled. One participant was excluded during the study as he did not fill in the required surveys. Data from 11 participants who completed the study were used for this analysis, who provided detailed information on their drug-related behavior, STI preventions measures and consent when having sex during 63 unique chemsex sessions.

The participants age ranged between 25 and 59 years old. The educational level of the participants was diverse and ranged from: no secondary school diploma (*n* = 2), secondary school diploma (*n* = 3), professional bachelor (*n* = 3), and master (*n* = 3). The vast majority of participants (*n* = 10) were full-time employed. Most participants participated in chemsex on a weekly (*n* = 5) or monthly (*n* = 4) basis during the three months prior to their enrollment in the study (Table 1).

**Table 1:** Characteristics of the study participants *n* = 11.

Variable	Categories	Number of participants
Age		
	25–29	3
	30–39	3
	40–49	2
	50–59	3
		
Educational status	No secondary school diploma	2
	Secondary school diploma	3
	Bachelor	3
	Master	3
	PhD	0
Employment status	Unemployed	1
	Employed (fulltime)	10
	Retired	0
Frequency chemsex	Daily	0
	More than once per week	1
	Weekly	5
	Monthly	4
	Less than once a month	1

### 2.4. Measures

Chemsex risk behavior was assessed via a risk-taking behavior questionnaire. This questionnaire was based on the chemsex-related risk behaviors that resulted from the needs assessment as part of the first consultation round in the app development process. The health problem of ‘participating in chemsex’ was conceptualized through previous research (literature study), a brainstorm with a large group of stakeholders, project team and advisory board, as well as via own research (in-depth interviews) (11).

The risk-taking behavior questionnaire contained 14 questions, divided into two overarching categories: high-risk drug use and high-risk sexual practices. For this analysis, we select answers on ten questions reflecting seven variables: (1) which drugs participants use, (2) duration of chemsex session, (3) dosing products safely, (4) avoiding STI transmission, (5) experiencing negative effects (during and after chemsex), (6) experiencing peer pressure (sexual and drug-related), and (7) consent (reciprocal). The complete ‘risk-taking behavior questionnaire’, and how we computed the answers for this analysis is described in Supplementary Table S1.

The participant was asked to complete the questionnaire at least twice during the study period. Each participant completed the questionnaire in the Budd app the day after having had participated in a chemsex session.

### 2.5. Statistical analysis

For this analysis, data are descriptive only, no statistical analysis was carried out.

### 2.6. Ethical statement

Ethical clearance was obtained from the Institutional Review Board of the Institute of Tropical Medicine in Antwerp (reference 1520/21; September 8th, 2021).

## 3. Results

In total, 11 participants provided information on 63 chemsex events. Participants reported behavior when participating in chemsex during a period of median 11 weeks and 5 days (ranging between 8 weeks, 5 days and 13 weeks). In this period, participants attended a mean of 5.7 chemsex sessions (median 4), ranging between 2 and 11 sessions. The mean duration of a chemsex session was 17.5 h (median 13 h), ranging between 2 and 48 h per session. Per session, participants used a mean of 3.5 different substances (median 4; range: 1–7). During three events (5%) one drug was used. In 95% of the events, different drugs were combined (‘polydrug use’).

Below, in Table 2, we present the results from the substances used during the chemsex sessions in the study, from the perspective of the used substance (which product is used how many times?; Table 2) (4, 13).

**Table 2:** Frequency of use, per product.

Product	Number of occasions used (%)
Category 1	
GHB/GBL	47 (74.6)
3MMC	44 (69.8)
Crystal meth	8 (12.7)
Mephedrone	8 (12.7)
Category 2	
Amphetamine	30 (47.6)
Ketamine	15 (23.8)
Ecstasy/MDMA	11 (17.5)
Cocaine	9 (14.3)
Category 3	
Poppers	26 (41.3)
Alcohol	17 (27.0)
Weed/hash	8 (12.7)
Total	223

We additionally created a table where we present the results from the substances used during the study from the perspective of the participant (which participant used which product?). We categorized the substances according to the review article by Strong and colleagues (4). We included the cathinone 3MMC, also referred to as ‘metaphedrone’, in category 1 as it is very similar to mephedrone (4MMC) (14). We add this table as Supplementary Table S2.

In Table 3, we present the detailed behaviors with regards to dosage of substances, STI prevention measure people take, experienced peer pressure (both sexual and substance-related pressure) and reciprocal sexual consent during chemsex sessions. With regards to the participants, we categorize a specific behavior if the participant reported this behavior at least once. This does not imply that this participant reports this behavior for every chemsex session he has attended.

**Table 3:** Reported behaviors, number of participants, and events.

		Participants (%)	Events (%)
Dosage and impact	Unsafe dosage	9 (81.8)	31 (49.2)
	Negative impact *during* event	7 (63.6)	21 (33.3)
	Negative impact *after* event	8 (72.7)	21 (33.3)
STI prevention	No preventive measures	7 (63.6)	20 (31.8)
Peer pressure	Peer pressure sex	9 (81.8)	31 (49.2)
	Peer pressure chems	9 (81.8)	36 (57.1)
Consent	Personal boundaries were not respected by other people	9 (81.8)	24 (38.1)
	I did not respect other peoples’ boundaries	9 (81.8)	19 (30.2)

## 4. Discussion

This study is the first where men who engage in chemsex systematically report their behavior for each separate chemsex event during a specified period of time. Although the number of participants in the study is too small to make absolute statements and their profile is heterogeneous, the information on the total number of chemsex sessions (*n* = 63) is considerable.

In our study, the two most commonly used products were GHB/GBL (used during 75% of the events) and the cathinone 3MMC (70%). The latter, also referred to as ‘metaphedrone’ is related to mephedrone (4MMC), which falls first category described above (4), as well as within the ‘narrow’ chemsex definition from the UK (1). Although in recent years this narrow definition has been broadened (to other substances), GHB/GBL and cathinones seem to remain the ‘core’ of chemsex use in our studied population, together with crystallized metamphetamine (‘crystal meth’). Metaphedrone (3MMC) seems to have replaced mephedrone when the latter became illicit. When we sum both cathinones mephedrone and 3MMC, it becomes the most commonly used drug (*n* = 52; used during 83% of the events). When considering the ‘number of participants’ as denominator (instead of the number of events), all participants reported the use of at least one ‘category 1’ substance: 2 participants report the use of one of these substances (18%), 7 used two (64%), and 2 (18%) all three of the ‘category 1’ drugs (GHB/GBL, 3MMC/4MMC, crystal meth).

Although the number of participants in our study is small, our findings are in line with results from recent qualitative and cross-sectional studies in our surrounding countries. Cathinones were found to be the most commonly used drug during chemsex research in France, followed by GHB and cocaine (7). In a study in the UK, mephedrone was the most common reported drug, followed by GBL and crystal metamphetamine (13). In a study in The Netherlands, Ecstasy/MDMA was most reported drug, followed by GHB (15). The frequent use of mephedrone and Ecstasy/MDMA may not be surprising as their effect is similar: alertness, feeling more empathic, and a positive effect on sexual desire (14).

In other European countries, methamphetamine (Spain), GHB/GBL (Norway, Spain, The Netherlands), and cocaine (Norway, France and Italy) were reported as frequently used drugs in a sexual context (16–18). Polydrug use is also very prevalent in our study: all participants reported the use of more than one substance, during 95% of all events. This is higher compared to studies in The Netherlands, United Kingdom and France (7, 15, 19, 20).

While being under the influence of drugs, chemsex participants seem to pose multidimensional ‘risky’ behavior: drug-related, related to their sexual health and related to their social situation. By crossing boundaries, people may experience adverse impact from their chemsex participation. The majority (9/11) reported their own boundaries were crossed, resulting in non-consensual sex; the same number of participants reciprocally did not respect others’ boundaries (9/11). This aspect of consent is particularly concerning, and requires more attention in chemsex research as findings show that sex without consent (or sexual violence) is reported in different studies. In the Netherlands, 58 of 273 men who participate in chemsex (21.1%) reported a non-consensual sexual experience in the past 5 years (21). In studies in the United Kingdom, the United States and Germany, the number of respondents who reported sexual violence and sex without consent was even more prevalent, with percentages between 43 and 48%. Moreover, participants who engaged in chemsex were up to 12.5 times more likely to experience sexual violence than their counterparts who did not engage in chemsex (22–24).

The majority of participants in the study reported dosing the substances ‘unsafe’, and experienced peer pressure and negative impact, including physical, psychological and social health harms, during and after the chemsex session. Related to their sexual health, the majority experienced peer pressure, and reported not having taken necessary preventive measures on at least one occasion. Of course, not every participant experiences all aspects (e.g., non-consensual sex, peer pressure) during each and every event. Nevertheless, peer pressure is experienced with regards to drug use during 57% of the events, peer pressure for sex in 49% of the events. Unsafe dosage is also reported in almost half of the events (49%). All other aspects (experiencing negative impact during and after the event, no preventive measures taken, and not respecting personal boundaries in two directions) are reported in 30–38% of the events (data not shown). These results confirm that, although similarities are obvious (e.g., polydrug use), chemsex can be experienced differently (e.g., frequency of participation, duration of a chemsex session, substances used).

We can identify some limitations in the study: we enrolled a convenience sample, thereby insufficiently controlling for participation bias. Moreover, the limited number of participants implies that the findings from this study cannot be generalized. Lastly, the limited number of variables assessed may obscure a multi-faceted picture of one’s individual situation and experiences. However, as participants were requested to provide information repeatedly, we balanced the comprehensiveness of the questionnaire with participants’ repetitive efforts to provide the same information. Despite these limitations, our study provides a snapshot of drug related behavior, sexual behavior and consent among a group of men engaging in chemsex using a study design that prevents recall bias. By assessing behavior during multiple chemsex sessions, ‘within-person’ variability can be clarified (one person can do different things during different chemsex sessions). This clarification may give insight in personal, emotional and contextual vulnerabilities which can be tackled during an individual care and support trajectory.

## 5. Conclusion

We consider a better insight in how people behave during chemsex events an important research question. First, to help in closing the scientific knowledge gap on the actual behavior during chemsex. The development of the Budd-mobile health intervention (10) was scientifically supported via the Intervention Mapping Protocol (25). Secondly, and potentially more relevant than the scientific knowledge, we hope that our findings support the improvement and optimization of care and support for people who engage in chemsex.

## Data availability statement

The raw data supporting the conclusions of this article will be made available by the authors, without undue reservation.

## Ethics statement

The studies involving human participants were reviewed and approved by Institutional Review Board of the Institute of Tropical Medicine in Antwerp (reference 1520/21; September 8th, 2021). The patients/participants provided their written informed consent to participate in this study.

## Author contributions

TP and CH collaborated equally in the set-up, execution of the study, analysis of the results, and writing the manuscript. EF, KP, and HV were involved in setting up and executing the study and provided valuable feedback on the manuscript. PV provided statistical and methodological advice in the set-up and analysis of the study and provided feedback on the manuscript. LA was involved in the enrolment of the participants and provided valuable feedback on the manuscript. All authors contributed to the article and approved the submitted version.

## Conflict of interest

The authors declare that the research was conducted in the absence of any commercial or financial relationships that could be construed as a potential conflict of interest.

## Publisher’s note

All claims expressed in this article are solely those of the authors and do not necessarily represent those of their affiliated organizations, or those of the publisher, the editors and the reviewers. Any product that may be evaluated in this article, or claim that may be made by its manufacturer, is not guaranteed or endorsed by the publisher.

## Supplementary material

The Supplementary material for this article can be found online at: https://www.frontiersin.org/articles/10.3389/fpubh.2023.1160087/full#supplementary-material
