# Supplementary material for: Drug behaviors, sexually transmitted infection prevention, and sexual consent during chemsex: insights generated in the Budd app after each chemsex session
Source: Front Public Health. 2023 May 18;11:1160087. doi: 10.3389/fpubh.2023.1160087 (PMC10234121; doi:10.3389/fpubh.2023.1160087)
Supplement: Supplementary file 1 [file Data_Sheet_1.PDF]

Supplementary Table 1: Risk Behavior Questionnaire

**Theme**	**Variable**	**Question**	**Answering categories**	**Computed**
General	Nutrition	I ate sufficiently during the chemsex date/party	Five-point Likert scale: *strongly disagree, disagree, neither agree nor disagree, agree, strongly agree*	Not computed for this analysis
	Hydration	I drank enough non-alcoholic beverages during the chemsex date/party	Five-point Likert scale: *strongly disagree, disagree, neither agree nor disagree, agree, strongly agree*	Not computed for this analysis
	Helping others	I helped others when they were feeling not well (if applicable)	Five-point Likert scale: *strongly disagree, disagree, neither agree nor disagree, agree, strongly agree*	Not computed for this analysis
Drugs	Drugs used	I took the following chems during the chemsex date/party	*Alcohol, GHB/GBL ('G'), Cocaine ('Coke'), Amphetamine ('Speed'), Crystallized methamphetamine ('TINA', 'Crystal Meth'), Mephedrone ('4-MMC'), 3MMC, Ecstasy/MDMA, Ketamine, Weed/hash, Poppers, Other*	NA
	Dosing safely	I dosed as safely as possible	Five-point Likert scale: *strongly disagree, disagree, neither agree nor disagree, agree, strongly agree*	Unsafe dosage: *strongly disagree, disagree*
	Experiencing negative effects	I experienced unwanted effects from the chems during the date/party	Five-point Likert scale: *strongly disagree, disagree, neither agree nor disagree, agree, strongly agree*	Experienced negative effects during chemsex: *agree, strongly agree*
		I experienced unwanted effects from the chems after the date/party	Five-point Likert scale: *strongly disagree, disagree, neither agree nor disagree, agree, strongly agree*	Experienced negative effects after chemsex: *agree, strongly agree*
	Peer pressure (drugs)	Other people present have tried to influence me to	Five-point Likert scale: *strongly disagree, disagree,*	Experienced drug-related peer

		take certain chems or doses	*neither agree nor disagree, agree, strongly agree*	pressure: *agree, strongly agree*
Sex	Duration of chemsex session	The number of hours I spent at the chemsex date/party	Insert number of hours	NA
	Medication/PrEP	Did I take my medication/PrEP correctly? (if applicable)	Yes / No	Not computed for this analysis
	Preventive measures to avoid STI transmission	I have taken measures to avoid STI transmission	Five-point Likert scale: *strongly disagree, disagree, neither agree nor disagree, agree, strongly agree*	'No preventive measures': *strongly disagree, disagree*
	Peer pressure (sex)	Other people present tried to convince me of certain sexual acts	Five-point Likert scale: *strongly disagree, disagree, neither agree nor disagree, agree, strongly agree*	Experienced sex-related peer pressure: *agree, strongly agree*
	Consent	I respected the boundaries of my sexual partner(s)	Five-point Likert scale: *strongly disagree, disagree, neither agree nor disagree, agree, strongly agree*	Partner did not consent for sex: *strongly disagree, disagree*
		My personal boundaries were respected by others	Five-point Likert scale: *strongly disagree, disagree, neither agree nor disagree, agree, strongly agree*	I did not consent for sex: *strongly disagree, disagree*
