# Supplementary material for: Drug behaviors, sexually transmitted infection prevention, and sexual consent during chemsex: insights generated in the Budd app after each chemsex session
Source: Front Public Health. 2023 May 18;11:1160087. doi: 10.3389/fpubh.2023.1160087 (PMC10234121; doi:10.3389/fpubh.2023.1160087)
Supplement: Supplementary file 2 [file Data_Sheet_2.PDF]

Supplementary table 2: Products used, and time attending chemsex sessions, per participant

**Participant number**	**Product category**	**Product**	**Number of times reported (%)**	**Hours median (mean; range)**
Participant 1 – 10 assessments	Category 1	GHB / GBL	10 (100)	
		3MMC	10 (100)	
		Mephedrone	2 (20)	
	Category 2	Amphetamine	6 (60)	
		Ketamine	6 (60)	
		Cocaine	5 (50)	
		Ecstasy / MDMA	2 (20)	
	Category 3	Poppers	1 (10)	
				48 (39; 12-48)
Participants 2 7 assessments	Category 1	Crystal meth	2 (28.6)	
	Category 2	Cocaine	2 (28.6)	
		Amphetamine	5 (71.4)	
	Category 3	Alcohol	7 (100)	
		Poppers	5 (71.4)	
		Weed / hash	5 (71.4)	
				15 (15; 8-20)
Participant 3 4 assessments	Category 1	GHB / GBL	4 (100)	
		3MMC	2 (50)	
		Crystal meth	1 (25)	
	Category 3	Poppers	2 (50)	
				2.5 (2.75; 2-4)
Participant 4 4 assessments	Category 1	3MMC	4 (100)	
	Category 2	Cocaine	1 (25)	
	Category 3	Alcohol	4 (100)	
		Poppers	2 (50)	
				12 (10.75; 6-13)
Participant 5 11 assessments	Category 1	GHB / GBL	10 (90.9)	
		3MMC	9 (81.8)	
	Category 2	Ecstasy / MDMA	2 (18.2)	
		Ketamine	1 (9.1)	
	Category 3	Poppers	6 (54.5)	
		Alcohol	2 (18.2)	
		Weed / hash	1 (9.1)	
				8 (8; 5-12)
Participant 6 8 assessments	Category 1	GHB / GBL	8 (100)	
		3MMC	8 (100)	
		Mephedrone	6 (75)	
	Category 2	Amphetamine	8 (100)	

		Cocaine	1 (12.5)	
	Category 3	Poppers	2 (25)	
				16.5 (16; 6/24)
Participant 7<br>2 assessments	Category 1	GHB / GBL	2 (100)	
		3MMC	2 (100)	
	Category 2	Ecstasy / MDMA	2 (100)	
	Category 3	Poppers	1 (50)	
				12 (12; 12-12)
Participant 8<br>2 assessments	Category 1	Crystal meth	2 (100)	
		3MMC	1 (50)	
	Category 3	Poppers	2 (100)	
				14 (14; 12-16)
Participant 9<br>9 assessments	Category 1	GHB / GBL	8 (88.9)	
		3MMC	5 (55.6)	
	Category 2	Amphetamine	8 (88.9)	
		Ketamine	8 (88.9)	
		Ecstasy / MDMA	2 (22.2)	
	Category 3	Alcohol	2 (22.2)	
				18 (20.8; 6-36)
Participant 10<br>2 assessments	Category 1	3MMC	2 (100)	
		GHB / GBL	1 (50)	
	Category 2	Amphetamine	2 (100)	
		Ecstasy / MDMA	1 (50)	
	Category 3	Alcohol	2 (100)	
		Weed/hash	1 (50)	
		Poppers	1 (50)	
				23.5 (23.5; 11-36)
Participant 11<br>4 assessments	Category 1	GHB / GBL	4 (100)	
		Crystal meth	3 (75)	
		3MMC	1 (25)	
	Category 2	Ecstasy / MDMA	2 (50)	
		Amphetamine	1 (25)	
	Category 3	Poppers	4 (100)	
		Weed / hash	1 (25)	
				9 (13; 8-26)
